# Impact of the COVID-19 pandemic on orthopedic trauma workload in a London level 1 trauma center: the “golden month”: The COVid Emergency Related Trauma and orthopaedics (COVERT) Collaborative

**DOI:** 10.1080/17453674.2020.1783621

**Published:** 2020-06-23

**Authors:** Chang Park, Kapil Sugand, Dinesh Nathwani, Rajarshi Bhattacharya, Khaled M Sarraf

**Affiliations:** Imperial College Healthcare NHS Trust & North West London Major Trauma Centre, London, UK

## Abstract

Background and purpose — The COVID-19 pandemic has been recognized as an unprecedented global health crisis. This is the first observational study to evaluate its impact on the orthopedic workload in a London level 1 trauma center (i.e., a major trauma center [MTC]) before (2019) and during (2020) the “golden month” post-COVID-19 lockdown.

Patients and methods — We performed a longitudinal observational prevalence study of both acute orthopedic trauma referrals, operative and anesthetic casemix for the first “golden” month from March 17, 2020. We compared the data with the same period in 2019. Statistical analyses included median (median absolute deviation), risk and odds ratios, as well as Fisher’s exact test to calculate the statistical significance, set at p ≤ 0.05.

Results — Acute trauma referrals in the post-COVID period were almost halved compared with 2019, with similar distribution between pediatric and adult patients, requiring a significant 19% more admissions (RR 1.3, OR 2.6, p = 0.003). Hip fractures and polytrauma cases accounted for an additional 11% of the modal number of injuries in 2020, but with 19% reduction in isolated limb injuries that were modal in 2019. Total operative cases fell by a third during the COVID-19 outbreak. There was a decrease of 14% (RR 0.85, OR 0.20, p = 0.006) in aerosol-generating anesthetic techniques used.

Interpretation — The impact of the COVID-19 pandemic has led to a decline in the number of acute trauma referrals, admissions (but increased risk and odds ratio), operations, and aerosolizing anesthetic procedures since implementing social distancing and lockdown measures during the “golden month.”

## The global impact of COVID-19

The novel coronavirus SARS-COV-2 (COVID-19) was first reported in December 2019 with the first patient hospitalized in the city of Wuhan, China (Wu et al. 2020). By mid-March 2020 the outbreak affected over 190 countries with over 450,000 cases and over 20,000 deaths, thus being declared a pandemic and a global public health emergency by the World Health Organization (2020). On January 24, 2020 Europe reported its first case followed by a case in the United Kingdom (UK) 5 days later (Spiteri et al. 2020). Such a pandemic is an unprecedented event, and governments have had to enact firm social distancing and lockdown measures in an attempt to mitigate further viral transmission (Anderson et al. 2020) in order to reduce morbidity and mortality.

## British response to the pandemic

The English government responded by implementing social distancing measures on the March 16, 2020 in an attempt to reduce the rate of transmission and therefore the demands on the National Health Service (UK Government 2020a). This was followed a week later by more stringent measures, commonly referred to as a societal “lockdown” (UK Government 2020b). As of March 23, 2020, all members of the public were required to stay at home except for limited purposes and this ruling received Royal Assent by March 26 within the rest of the UK. Furthermore, all public gatherings of more than 2 people and non-essential businesses were suspended. In response to the NHS emergency declaration (National Health Service England 2020), the Royal College of Surgeons (2020) and the British Orthopaedic Association (2020) both issued statements and guidelines for delivering emergency trauma and orthopedic care during the COVID-19 outbreak. The phenomenon of a reduction in trauma burden due to such social distancing measures has been described by Stinner et al. (2020), as well as the potential impact of COVID-19 on operative capacity and pathways. There has been little to explore on how COVID-19 affects the etiology of trauma referral workloads and the operative casemix.

We evaluated the impact of the COVID-19 pandemic at a central London level 1 trauma center, also known as a Major Trauma Centre (MTC), evaluating the trends of acute orthopedic trauma referral caseload and operative casemix before (2019) and during (2020) the COVID-19 lockdown (i.e., the “golden” month period starting from March 17).  

## Methods

### Patient sampling

All acute referrals, operative notes, inpatient medical records, and discharge summaries were accessed using the electronic medical system.

### Study period

The study period was from the start of social distancing on the morning of March 17, 2020 to April 15, 2020, which also encompasses the morning following more firm “lockdown” measures on March 24, 2020. This was compared with the same 4-week interval in March–April 2019 prior to any COVID-19 related measures to compare its impact 1 year apart.

### Inclusion criteria

The study criteria comprised all acute orthopedic trauma referrals presenting to the Emergency Department of a busy level 1 trauma center (North West London Major Trauma Centre, UK) during the stated intervals 1 year apart, and all orthopedic trauma cases that required an operation, including those from acute orthopedic trauma referrals, within the stated intervals 1 year apart. Those patients listed for an operation prior to the period of data collection were included in the final analysis. We adhered to the STROBE guidelines.

### Exclusion criteria

Any cases referred internally from other specialties for trauma and orthopedic advice and input, as well as referrals from any external center asking for tertiary advice, were excluded from further analysis. Any patient with a postoperative complication arising in the period prior to the data collection were excluded. For operative trauma cases, those undergoing spinal procedures were excluded as the service is delivered jointly by the neurosurgery service. With respect to infections, all acute and chronic surgical site infections (SSI) and non-SSIs were excluded from the final analysis. All non-urgent semi-elective procedures were excluded from analysis as well, to avoid inaccurate assessment of the impact of any social distancing measures on trauma workloads. Routine elective orthopedic cases were excluded.

### Data points

Demographics including age, sex, and ASA grades were recorded for all patients. Injury characteristics were recorded, including the anatomical location and whether the injury was open or closed. The mechanism of injury was categorized and whether the patient presented as a trauma call. The nature of the operative procedures and the anesthetic techniques were recorded. Patients undergoing multiple procedures were recorded for every episode when they were taken to theatre.

### Statistics

All the data were recorded, anonymized, and verified by 2 authors for their accuracy. The median (median absolute deviation) was calculated for both age and ASA grade. Both risk and odds ratios were calculated as well as a Fisher’s exact test for statistical significance, defined as p ≤ 0.05.

### Ethics, funding, and potential conflicts of interest

No formal ethical approval was required as these were audit data. The study was registered and approved with the Trust’s audit department. No identifiable patient data have been kept or reported. This study required no internal or external funding. The authors have no conflict of interests to declare. 

## Results

There were no missing data, as all data points were extracted from electronic patient records (Figure).

### Pre-COVID era

For the pre-COVID period in 2019 there were 193 new referrals. 106 (55%) were male. 31 patients were excluded, which left 162 patients in the pre-COVID period who were acutely referred. 90 (56%) were male. 135 (83%) patients were adults (> 18 years old) (Figure).

### Post-COVID era

For the post-COVID period in 2020 there were 94 referrals (53% of those in 2019). Sex was split equally.

### Demographics

7 patients were excluded, which left 87 new acute trauma referrals in the post-COVID period (Figure). 44 (51%) patients were male. 75 (86%) patients were adults.

Results have been tabulated as acute referrals, categorized as all referrals, adult referrals, and pediatric referrals between the 2 years (Table 1). Table 2 reflects the operative casemix.

**Table 1.** Referrals between pre- and post-COVID

	Acute trauma referrals		Adults		Paediatric	
	Pre-COVID	Post-COVID	Pre-COVID	Post-COVID	Pre-COVID	Post-COVID
	n = 162	n = 87	n = 135	n = 75	n = 27	n = 12
Demographic						
** **Male	90	43	73	38	17	6
** **Female	72	43	62	37	10	6
** **Age ^a^	47 (26)	50 (24)	54 (21)	56 (23)	9 (4)	9.5 (5.5)
** **ASA ^a^	1 (0)	2 (1)	2 (1)	2 (1)	1 (0)	1 (0)
Injury						
** **Upper limb	49	22	34	14	15	8
** **Lower limb	54	22	47	20	7	2
** **Hip	14	17	14	17	0	0
** **Pelvis	8	2	7	2	1	0
** **Polytrauma	18	17	18	1	0	0
** **Infection	15	7	12	5	3	2
** **Other	4	0	7	0	1	0
Mechanism of injury						
** **Sporting	18	2	11	2	7	0
** **Fall	80	50	67	44	14	6
** **Fall from height > 1.5 m	8	9	8	8	0	1
** **Road traffic collision	25	13	24	12	1	1
** **Pathological	6	1	3	0	0	1
** **Other	25	12	20	9	5	3
Open injury	23	17	21	15	2	2
Trauma call	44	25	42	24	2	1
Operative requirement	76	48	66	43	10	5

**^a^**Median and (median absolute deviation).

ASA: American Society of Anesthesiologists.

**Table 2.** Operative trauma casemix between pre- and post-COVID

	Operative trauma cases only	
	Pre-COVID	Post-COVID
	n = 90	n = 63
Demographic		
** **Male	49	39
** **Female	41	24
** **Age ^a^	43.5 (19)	50 (20)
** **ASA ^a^	1 (0)	2 (1)
Injury		
** **Upper limb	21	12
** **Lower limb	30	12
** **Hip	16	14
** **Pelvis	2	1
** **Polytrauma	17	16
** **Infection	2	5
** **Other	2	3
Mechanism of injury		
** **Sporting	7	0
** **Fall	36	33
** **Fall from height > 1.5 m	4	6
** **Road traffic collision	25	15
** **Pathological	2	0
** **Other	6	9
Open injury	28	20
Trauma call	36	28
Operation		
** **Total	91	67
** **MUA	4	3
** **External fixator	7	5
** **Frame	1	2
** **Removal of metal	2	1
** **Soft tissues/other	12	10
** **Percutaneous wiring	2	2
** **ORIF	34	23
** **Intramedullary device	16	13
** **Dynamic hip screw	8	4
** **Hemiarthroplasty	5	4
Anaesthetic method		
** **General anaesthesi (GA)	78	46
** **Spinal	3	10
** **GA + spinal	7	5
** **Block	0	2
** **Local	2	0

**^a^**Median and (median absolute deviation),

ASA: American Society of Anesthesiologists,

MUA: manipulation under anaesthesia.

### Prevalence, risk, and odds ratios

Table 3 outlines the prevalence and prevalence odds ratios alongside their 95% confidence intervals (CI) and statistical significance. The risk ratio is synonymous with the prevalence ratio. There was no statistically significant difference in the number of trauma calls and adult versus pediatric acute trauma referrals between the 2 years. On closer inspection, even though just over half the acute orthopedic trauma referrals were made in the post-COVID period, there was a greater proportion of acute presentations referred, a 30% (RR 1.3, CI 1.1–1.5) increased prevalence of admission with the odds of admission increased by 156% (OR 2.6, CI 1.4– 4.7). Hence, the threshold for referral was much lower and these patients were more in need of inpatient medical care in spite of admission of trauma patients being discouraged to reduce viral transmission and to uphold patient safety.

**Table 3.** Risk and Odds ratios (95% CI)

	Pre vs Post COVID		Fisher’s
	RR	OR	p-value
Acute referrals requiring admission	1.3 (1.1–1.5)	2.6 (1.4–4.7)	0.003
Acute referrals requiring surgery	1.2 (0.9–1.5)	1.4 (0.8–2.4)	0.2
Consultant-led operations	1.2 (1.0–1.4)	2.3 (1.0–5.4)	0.05
Operations requiring GA (± spinal)	0.9 (0.8–1.0)	0.2 (0.1–0.7)	0.006
Adult vs paediatric acute referrals	1.0 (0.9–1.2)	1.3 (0.6–2.6)	0.6
Trauma calls	1.0 (0.6–1.5)	1.0 (0.5–1.7)	1
Sporting injuries from acute referrals	0.2 (0.1–0.9)	0.2 (0.0–0.8)	0.01

Although a greater number of the acute referrals required surgery, this was ultimately not statistically significant (RR 1.2, CI 0.9–1.5, OR 1.4, CI 0.8–2.4). Nevertheless, if a patient did require surgery during the COVID outbreak, there was a 131% (OR 2.3, CI 0.99–5.4) increased odds that the operation would be consultant-led (either as primary surgeon or scrubbed in to supervise, as opposed to being unscrubbed) with a 19% increased prevalence of personal involvement compared with 2019. As expected, every attempt was made to minimize reliance on aerosolizing anesthetic procedures wherever possible in order to reduce viral transmission, load, and exposure to the healthcare staff. In 2020, COVID significantly decreased the prevalence of GA (±spinal) by 15% (RR 0.85, CI 0.75–0.96) as well as decreasing the odds of receiving an aerosolized anesthetic procedure by 80% (OR 0.20, CI 0.06–0.65).

## Discussion

### A shift in clinical practice

There was a notable difference between the number of acute referrals and the operative casemix between the time intervals 1 year apart pre- and post-COVID in a London level 1 trauma center. There was a substantial decrease in the number of relevant acute trauma referrals (without a statistically significant difference between age and sex but significantly fewer sporting injuries), number of operations (without significant difference between mechanisms of injury and type of surgery or technique) with a lower number of aerosolizing anesthetic procedures (with significantly less risk and odds ratios). This reduction is likely to have been a direct consequence of the social distancing measures implemented on a national scale.

### Effect of local, regional, and nationwide service reconfiguration

Post-COVID there has also been a reduction in external referrals as compared with 2019, representing the effects of the major service reconfiguration with disbanding of the elective, private, and inpatient practice to cater for the increased space requirement to host acute COVID patients pre- and post-ITU treatment. To reduce the risk of virus transmission and reduce the demands on services, some injuries previously treated at the level 1 trauma center were being treated at level 2 trauma units, a change of practice supported by the British Orthopaedic Association (2020). There are 22 equivalent level 1 trauma centers in England out of 152 acute specialist trusts with a further 5 pediatric specific units (National Health Service England 2016). Within London there are 4 level 1 trauma centers of which our center is 1 of the largest. In the pre-COVID period, level 1 trauma centers would exclusively provide care for polytrauma, complex and open injuries. However, these may now be expected to be managed at smaller level 2–4 trauma units (i.e., district general hospitals [DGH]) as highlighted by Morgan et al. (2020). Some injuries such as Gustilo-Anderson type 3 injuries, polytrauma, complex intra-articular fractures, and those requiring cross-specialty expertise from plastic and vascular surgeons, will continue to require treatment at a level 1 trauma center as the specialist skill may be unavailable at many level 2 trauma units. However, as level 1 trauma centers are present in less than a fifth of all English acute NHS trusts, the COVID pandemic may alter the expectations and the role of level 1 trauma centers in the future, especially in the treatment of those injuries not requiring cross-specialist input or complex management in the first instance.

### Demographic and injury pattern

The comparison of demographics of acute trauma referrals is mostly similar between the 2 periods, as seen in Table 1. There is a near equal split in sex in 2019 and an exact split in 2020. Similarly, in 2019, 83% of referrals were adults compared with 86% in 2020, with a higher median ASA grade (2) to signify sicker patients.

Pre-COVID in 2019, the most common injury pattern for acute referrals was lower limb injuries at 33%. This was followed by upper limb injuries (30%) and together they accounted for nearly two-thirds of all referrals. Post-COVID, both upper and lower limb injuries are still the most common injury but combined they accounted for just 50% of all referrals. Hip fractures and polytrauma (often from road traffic collisions and high-energy injuries) patients, however, accounted for an increased proportion of acute referrals, each accounting for 19% of cases.

### Hip fractures (HF)

Usually the result of low-energy falls, HFs often occur indoors, in the garden, and within the property, and the incidence may not be directly impacted by the social distancing measures that were implemented. Therefore, it would be expected that the number of HF referrals would be consistent, as demonstrated by 17 HF referrals in 2020, an increase from the 14 in 2019. HF referrals reflected a higher percentage of all acute referrals during the pandemic study period when compared with 2019 (i.e., 19% vs. 9%). Yet there was no significant change in risk or odds ratios of HF between the 2 years.

### Polytrauma

Conversely, polytrauma occurs as a consequence of high-energy injuries. This may often be as a result of a road traffic collision (RTC) or fall from a height greater than 1.5 m. RTC rates have not changed significantly following social isolation advice and may account for some of the greater proportion of polytrauma observed. There has been an increase in those falling from a height greater than 1.5 m in 2020 of 10% as compared with 5% in 2019. This could be due to the construction industry being exempt from the lockdown or people spending more time at home committing to home improvements or do-it-yourself tasks.

### Mechanism of injury (MOI)

The breakdown of the mechanism of injury has remained largely the same pre- and post-COVID as seen in Tables 1–2. A fall from less than a 1.5 m height still accounts for the majority of cases in 2020 at 58%, compared with 49% in 2019. Nevertheless patients, especially the geriatric community, will continue to suffer from low-energy falls despite the social isolation, be it within their homes, from simple falls and trips, and this may explain the overall consistency.

### Sporting injuries

There has been an 89% reduction in acute referrals due to sporting injuries and a 100% drop in the MOI in the operative casemix 1 year apart (Table 2). This would be expected, as all group activities have been banned following social isolation and gyms are closed to reduce the risk of viral transmission. This significant decrease may represent the main etiology of the reduction in trauma referrals seen between the 2 periods (RR 0.21, CI 0.05–0.87; OR 0.2, CI 0.04–0.83), which would correlate closely with the government’s advice post-COVID.

### Road traffic collisions (RTCs)

Despite the significant reduction in personal vehicle use, there has been a consistent proportion of injuries seen following social isolation attributable to RTCs (Table 1) at 15% (n = 13) post-COVID compared with 15% in 2019. Although the roads are quieter, there have been concerns that, paradoxically, fewer vehicles may result in more RTCs due to speeding. The Metropolitan Police have described an increase incidence of speeding, with average speeds of 37 mph in some 20 mph zones, following social isolation (British Broadcasting Corporation 2020). Although the data represent a 40% decrease in the number of patients being admitted following an RTC, nevertheless this is some way from the total reduction of road use estimate of 70% (Carrington 2020).

### Operative cases

Accounting for all exclusions, the total number of operative cases has fallen by a third following COVID. A proportion of this is due to the ceasing of semi-elective operating. However, the reality of practice at our center is that the management of orthopedic injuries during the COVID era has not changed significantly. Non-urgent and elective procedures have been cancelled or postponed following national advice (National Health Service England 2020), but the decision to offer operative intervention is still, first and foremost, a decision based on clinical need, balancing risk and benefit to the patient. The key driving force behind the overall reduction in operative procedures performed in 2020 is the reduction in referral volume (Table 2) and not due to an altered threshold of operative intervention. Indeed, we currently do not anticipate any fracture complication or secondary intervention required as being directly due to any altered management decision during the COVID period.

### Anesthetic choice

There was a preference for performing non-aerosol generating procedures (AGPs) as the anesthetic methods in 2020 (19%) compared with 2019 (6%) as seen in Tables 2 and 3. There has been evidence that AGPs such as intubation for a GA increases the risk of healthcare work transmission with an increased viral load (Vannabouathong et al. 2020). As such, this change may represent a shift in an attempt to mitigate this risk. In order to avoid AGPs in the theatre setting, patients are encouraged to consent and agree to regional blocks including spinals, which in themselves also take longer to perform and have an effect compared with intubation for GA. Whereas 87% of total patients operated on pre-COVID had a GA, this was reduced to 73% post-COVID with an increase in regional blocks from 3% to 16% among all patients during the COVID period.

### Limitations and future studies

The limitations of this longitudinal observational study include analyzing 2x4-week snapshots 1 year apart, at a single-center London level 1 trauma center. This may not be representative of the national profile. There has been much literature on the benefits of large-volume centralization of orthopedic trauma to level 1 trauma centers, but this might need to be re-analyzed if smaller peripheral trauma units return to managing grade 1–2 Gustilo Anderson open fractures and simpler closed polytrauma. Further work is required to observe for trends in acute orthopedic referrals and orthopedic trauma surgical casemix as a result of the structural reconfiguration due to COVID. Bias was kept to a minimum and the date range between the two years was dictated by the evolution of the pandemic.

## Conclusion

The COVID-19 pandemic has had a unique impact on trauma and orthopedic care. Acute trauma referral rates have fallen, with fewer trauma procedures being performed since the implementation of the UK social distancing measures indicating a change in prevalence pre- and post-COVID. Every attempt has been made to substantially reduce the prevalence of aerosol-generating anesthetic procedures with general anesthesia and intubation. We recommend more work to investigate the phenomenon further and whether a similar pattern is seen across the UK.

**Figure:**
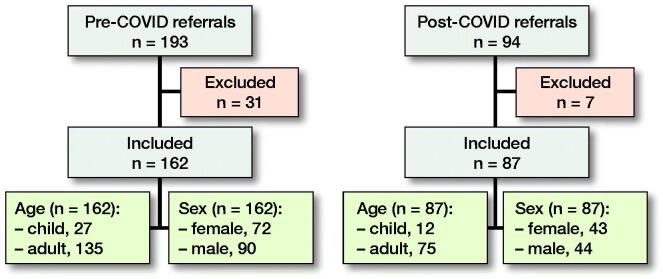
Demographic data pre- and post-COVID for acute referrals.
